# Enhanced mitochondrial glutamine anaplerosis suppresses pancreatic cancer growth through autophagy inhibition

**DOI:** 10.1038/srep30767

**Published:** 2016-08-01

**Authors:** Seung Min Jeong, Sunsook Hwang, Kyungsoo Park, Seungyeon Yang, Rho Hyun Seong

**Affiliations:** 1 Department of Biochemistry, College of Medicine, The Catholic University of Korea, 222, Banpo-daero, Seocho-gu, Seoul, 06591, Republic of Korea; 2 Institute for Aging and Metabolic Diseases, College of Medicine, The Catholic University of Korea, 222, Banpo-daero, Seocho-gu, Seoul, 06591, Republic of Korea; 3 School of Biological Sciences and Institute of Molecular Biology and Genetics, Seoul National University, Seoul 151-742, Republic of Korea

## Abstract

Cancer cells use precursors derived from tricarboxylic acid (TCA) cycle to support their unlimited growth. However, continuous export of TCA cycle intermediates results in the defect of mitochondrial integrity. Mitochondria glutamine metabolism plays an essential role for the maintenance of mitochondrial functions and its biosynthetic roles by refilling the mitochondrial carbon pool. Here we report that human pancreatic ductal adenocarcinoma (PDAC) cells have a distinct dependence on mitochondrial glutamine metabolism. Whereas glutamine flux into mitochondria contributes to proliferation of most cancer cells, enhanced glutamine anaplerosis results in a pronounced suppression of PDAC growth. A cell membrane permeable α-ketoglutarate analog or overexpression of glutamate dehydrogenase lead to decreased proliferation and increased apoptotic cell death in PDAC cells but not other cancer cells. We found that enhanced glutamine anaplerosis inhibits autophagy, required for tumorigenic growth of PDAC, by activating mammalian TORC1. Together, our results reveal that glutamine anaplerosis is a crucial regulator of growth and survival of PDAC cells, which may provide novel therapeutic approaches to treat these cancers.

Pancreatic ductal adenocarcinoma (PDAC), the most common type of pancreatic cancer, is highly lethal and has a poor prognosis^1^. The lethality of these refractory cancers stems from its late diagnosis as well as a propensity to rapid metastasize^2^,^3^. Moreover, PDAC has profound resistance to all forms of therapy, such as chemotherapy, radiotherapy and targeted agents^4^,^5^. Thus, the identification of new therapeutic targets for PDAC is a high priority.

The altered cellular energy metabolism is one of key features of cancer. To fulfill their energetic and synthetic needs, cancer cells reprogram their metabolic pathways, which supports growth and survival of cancer cells^6^,^7^. Because proliferating cells use precursors derived from tricarboxylic acid (TCA) cycle intermediates, replenishment of the mitochondrial carbon pool is essential for the maintenance of mitochondrial integrity. Glutamine (Gln), as an important nitrogen and carbon donor for cell, provides this mitochondrial anaplerosis^8^,^9^,^10^. In mitochondria, Gln is catabolized via glutaminase (GLS) to glutamate and ammonia (NH_4_^+^) and further converted to the TCA cycle intermediate α-ketoglutarate (αKG) via glutamate dehydrogenase (GDH) or transaminases to fulfill mitochondrial carbon pool. This incorporation of Gln-derived αKG into the TCA cycle is the major anaplerotic step in proliferating cells. Indeed, mitochondrial Gln metabolism is required for oncogene-induced tumorigenesis, and many cancer cells exhibit an enhanced mitochondrial Gln anaplerosis and increased levels of TCA cycle intermediates^6^,^8^.

Recent evidence demonstrates that PDAC cells rely on a non-canonical Gln pathway. In PDAC cells, Gln-derived glutamate is mainly converted to aspartate via a glutamic-oxaloacetic transaminase (GOT1) and then aspartate is transported into the cytoplasm to maintain the cellular redox homeostasis^11^. Oncogenic KRAS, serving a critical role in PDAC initiation and maintenance, mediates this reprogramming of Gln metabolism through the transcriptional regulation of GOT1 and GDH expression^11^. However, the role of canonical mitochondrial Gln metabolism in PDAC was not well elucidated.

Autophagy is the cellular destructive mechanism, allowing the recycling of unnecessary or dysfunctional components^12^,^13^,^14^. Whereas most cells exhibit low levels of autophagy which is induced in response to cellular stresses such as nutrient deprivation, DNA damage and accumulation of unfolded proteins, PDAC cells have elevated levels of autophagy under basal conditions^15^. Moreover, inhibition of autophagy results in tumor regression and an increase in survival in the PDAC mouse model, indicating that enhanced autophagy expression is essential for tumorigenic growth of PDAC.

In this study, we sought to probe the role of mitochondrial Gln metabolism in pancreatic cancer. We demonstrate that enhanced mitochondrial Gln anaplerosis markedly inhibits PDAC growth and induces apoptotic cell death by repressing cellular autophagy levels, identifying a new aspect of Gln metabolism in PDAC growth and survival.

## Results

### Enhanced mitochondrial Gln anaplerosis inhibits the growth of PDAC

Because PDAC cells are sensitive to Gln withdrawal and knockdown of GLS, the first enzyme of mitochondrial Gln metabolism, significantly attenuates their growth^11^, we hypothesized that mitochondrial Gln metabolism could serve as an important regulator of PDAC growth and survival. To test this idea, we examined whether the enhanced mitochondrial Gln anaplerosis supports tumorigenic growth of PDAC. As incorporation of αKG into the TCA cycle is the key step of anaplerosis, we used a cell membrane-permeable dimethyl-αKG (DMKG) to increase mitochondrial Gln metabolism. We first tested whether DMKG treatment supports mitochondrial anaplerosis in PDAC cells. Mitochondrial Gln catabolism is essential for cell viability in the absence of glucose^16^. Thus, GLS1 inhibition by bis-2-(5-phenylacetoamido-1,2,4-thiadiazol-2-yl)ethyl sulfide (BPTES)^17^ markedly increased cell death in glucose-free conditions. This cell death was rescued by the addition of DMKG (Supplementary Fig. 1a). Moreover, we found that intermediates of TCA cycle, such as succinate and malate, were elevated in DMKG treated cells compared to control cells (Supplementary Fig. 1b).

**Figure 1:**
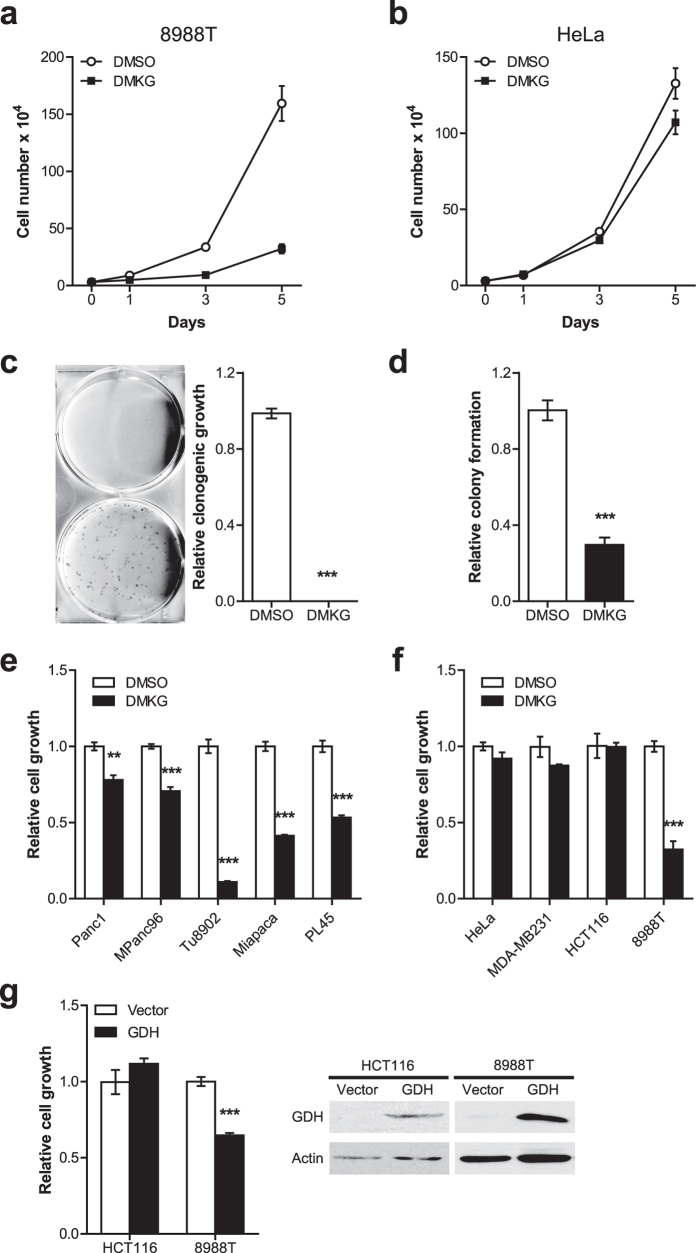
DMKG treatment or GDH overexpression repress PDAC growth. (**a,b**) Growth curves of 8988T (**a**) and HeLa (**b**) cells cultured in 6-well plates with or without DMKG (5 mM). Error bars, ±SD. (**c**) Clonogenic assays of 8988T cells with or without DMKG. Cells were cultured for 8 days and stained with crystal violet. Representative wells of the clonogenic growth experiment (left). The number of colonies was counted (right). (**d**) Soft agar assays of 8988T cells with or without DMKG. (**e**) Relative proliferation of PDAC cell lines (Panc1, MPanc96, Tu8902, Miapaca2 and PL45) with or without DMKG. (**f**) Relative proliferation of cancer cell lines (HeLa, MDA-MB231, HCT116 and 8988T) with or without DMKG. (**g**) Relative proliferation of HCT116 and 8988T cells stably expressing empty vector (Vector) or GDH. All error bars (except growth curves), ±SEM. **p < 0.01 and ***p < 0.001.

We next assessed PDAC growth in the presence of DMKG. To our surprise, DMKG treatment markedly repressed the growth of 8988T PDAC cells (Fig. 1a). To further validate the effect of DMKG on tumorigenic growth of PDAC cells, we assessed their clonogenic growth with or without DMKG. Addition of DMKG almost completely inhibited the clonogenic growth of 8988T cells (Fig. 1c). In line with these, similar results were observed by assessing their anchorage-independent growth of 8988T cells (Fig. 1d). Moreover, we confirmed the effects of DMKG treatment with multiple PDAC lines (Fig. 1e).

To test whether the growth inhibition by enhanced Gln anaplerosis is a common feature of cancer cells, we assessed cell growth of several cancer cells after DMKG treatment. Consistent with studies showing that DMKG had no harmful effect on cell proliferation and rather restored cell growth and the levels of TCA cycle intermediates upon Gln deprivation^18^,^19^, we found that DMKG treatment did not affect proliferation of cervical (HeLa), breast (MDA-MB231) and prostate (HCT116) cancer cell lines (Fig. 1b,f).

GDH is the primary driver of mitochondrial Gln metabolism and its overexpression increases intracellular levels of αKG^20^. Interestingly, it was shown that the expression of GDH is negatively regulated by oncogenic KRAS in PDAC cells^11^, implying that the repression of GDH might be required for PDAC growth. Given the importance of Gln anaplerosis in PDAC, we speculated that overexpression of GDH may inhibit PDAC growth. Importantly, we observed that GDH overexpression had a growth-suppressive effect on 8988T cells (Fig. 1g). However, GDH overexpression no further repressed cell growth when GLS1 is inhibited by BPTES (Supplementary Fig. 2), demonstrating that enhanced mitochondrial Gln metabolism by GDH is responsible for growth suppression of PDAC cells. Consistent with our previous results, we found comparable cell growth in control and GDH overexpressed HCT116 cells (Fig. 1g). Taken together, these data demonstrate that an enhanced mitochondrial Gln anaplerosis represses PDAC growth.

### Enhanced mitochondrial Gln anaplerosis induces apoptotic cell death in PDAC

**Figure 2:**
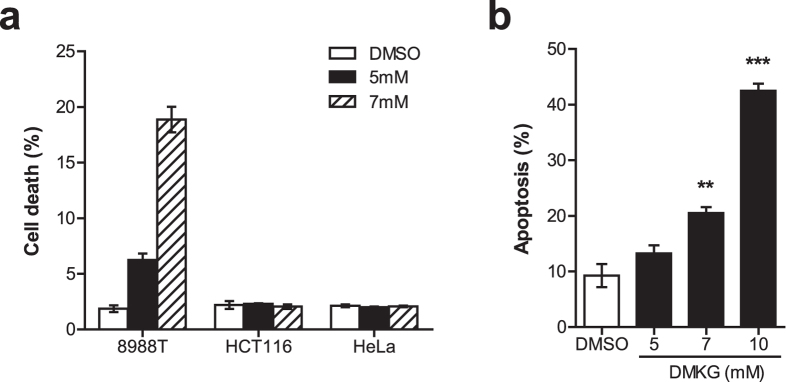
DMKG treatment induces cell death in PDAC. (**a**) Survival of 8988T, HCT116 and HeLa cells cultured with or without DMKG (5 or 7 mM). (**b**) Apoptotic cell death of 8988T cells cultured with indicated doses of DMKG treatment. All error bars ± SEM. **p < 0.01 and ***p < 0.001.

Our results indicate that mitochondrial Gln metabolism may be a key regulator of PDAC growth. Next, to examine its role in PDAC survival, we assessed the sensitivity of 8988T cells to DMKG. We found that DMKG treatment significantly induced cell death of 8988T cells in a dose-dependent manner, whereas there were no obvious changes in cell survival of other cancer cells, such as HCT116 and HeLa cell lines (Fig. 2a). When we examined whether the enhanced Gln anaplerosis induces apoptotic cell death in PDAC cells by using AnnexinV-staining, similar results were observed (Fig. 2b). Our data indicate that PDAC cells are significantly more sensitive to the enhanced Gln anaplerosis.

### Gln anaplerosis regulates autophagy in PDAC

**Figure 3:**
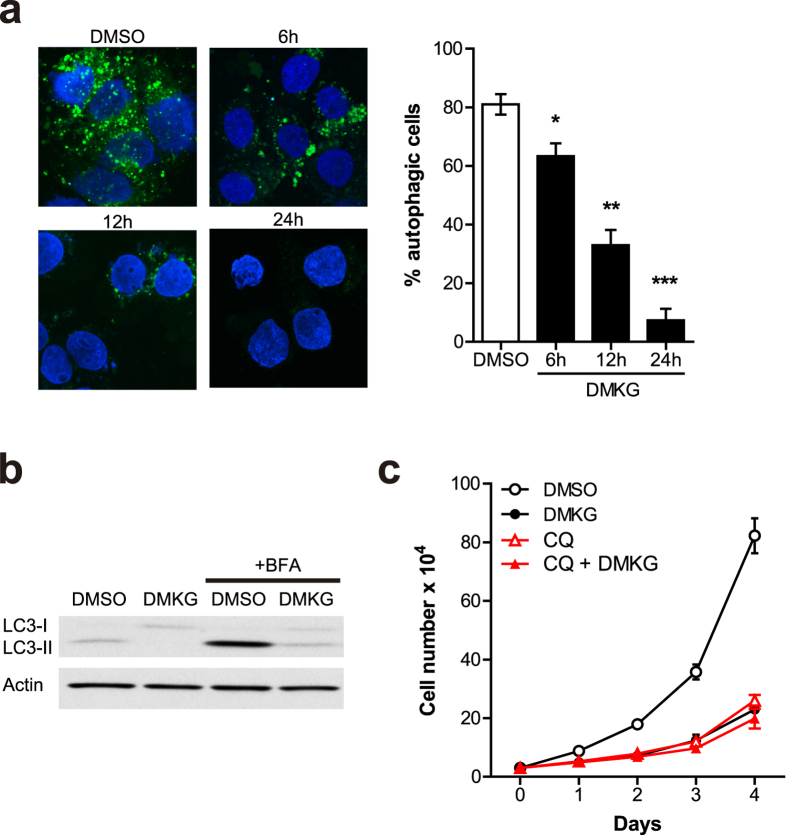
Gln anaplerosis inhibits autophagy in PDAC. (**a**) Aggregation of GFP-LC3 in 8988T cells treated with DMKG (5 mM) for the indicated times. Representative images of fluorescence microscopic analysis (left). The percentage of autophagic cells (defined as the presence of more than five autophagy foci) was quantified (right). (**b**) Endogenous LC3-II protein levels in whole-cell lysates from 8988T cells treated with or without DMKG. Where indicated, 200 nM of bafilomycin A (BFA) was present for 2 hr to inhibit the degradation of LC3-II. β-actin serves as a loading control. (**c**) Growth curves of control and chloroquine (100 μM) treated 8988T cells cultured in 6-well plates with or without DMKG. Cell number was measured every 24 hr for 4 consecutive days. Error bars, ±SD. All error bars ±SEM. *p < 0.05, **p < 0.01 and ***p < 0.001.

Autophagy is the well-coordinated destructive mechanism, allowing the orderly degradation and recycling of cellular components^21^. It was shown that pancreatic cancers require autophagy for growth and survival^15^. As recent evidence demonstrates that an increased glutaminolysis inhibits autophagy formation^20^, we hypothesized that Gln anaplerosis might regulate PDAC growth and survival by regulating autophagy levels. To test this idea, we assessed whether DMKG treatment represses cellular autophagy levels in PDAC cells. As has previously been shown^15^, we observed that 8988T cells exhibited high levels of autophagy under basal conditions (Fig. 3a), as measured by formation of GFP labeled microtubule-associated protein light chain 3 (LC3) puncta. Importantly, addition of DMKG was sufficient to inhibit autophagy in a time dependent manner (Fig. 3a). Notably, the ability of DMKG to inhibit autophagy was significantly attenuated in Gln deprived (0.1 mM) medium, indicating that the increased Gln anaplerosis is responsible for autophagy inhibition in PDAC cells (Supplementary Fig. 3). As further confirmation of the inhibition of autophagy, we determined the effect of DMKG treatment on levels of the lipidated form of LC3 (LC3-II). Addition of DMKG markedly reduced LC3-II expression in 8988T cells (Fig. 3b). We observed similar results in the presence of the lysosomal inhibitor bafilomycin A1 (BFA) to determine autophagic flux.

It was shown that inhibition of autophagy attenuates tumorigenic growth of PDAC cells^15^. To investigate the contribution of decreased autophagy to the growth inhibition of PDAC by enhanced Gln anaplerosis, we cultured 8988T cells in media containing chloroquine (CQ), an autophagy inhibitor, with or without DMKG treatment, and measured cell proliferation. We found that DMKG treatment had no further growth inhibitory effect in the presence of CQ (Fig. 3c). Taken together, these findings suggest that the repression of autophagy may contribute to the effects of enhanced Gln anaplerosis in PDAC.

### Gln anaplerosis regulates autophagy by activating mTORC1 signaling

**Figure 4:**
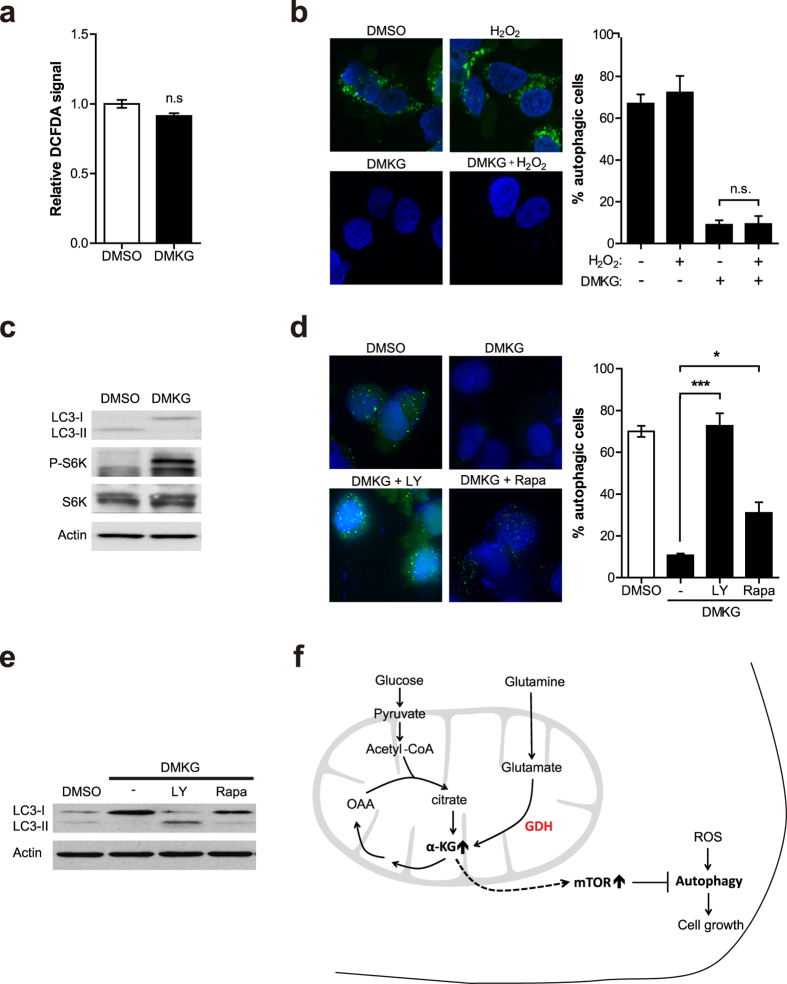
Gln anaplerosis represses the autophagy not by ROS regulation but by mTOR signaling in PDAC. (**a**) Dichlorofluorescin diacetate (DCFDA) was measured in 8988T cells treated with or without DMKG. (**b**) Representative images of GFP-LC3 in control or DMKG treated 8988T cells cultured with or without 0.5 mM H_2_O_2_ (left). The percentage of autophagic cells was quantified (right). (**c**) Immunoblot analysis of LC3-II, phosphor-S6K and S6K protein levels in whole-cell lysates from 8988T cells treated with or without DMKG. β-actin serves as a loading control. (**d,e**) Representative images of GFP-LC3 (**d**) and LC3-II protein levels (**e**) in control or DMKG treated 8988T cells cultured with DMSO, LY29004 (50 μM) or rapamycin (500 nM). β-actin serves as a loading control. (**f**) A proposed model illustrating the regulation of PDAC growth by Gln anaplerosis. All error bars ± SEM. n.s., not significant. *p < 0.05 and **p < 0.01.

To investigate the mechanisms underlying the inhibition of autophagy by mitochondrial Gln metabolism in PDAC, we examined several pathways known to regulate autophagy. Previous studies reported that autophagy is regulated by reactive oxygen species (ROS)^15^,^22^. In PDAC, the antioxidant treatment significantly reduced autophagy levels, whereas exogenous ROS could induce autophagy in non-transformed pancreatic cells^15^. It was also shown that GDH inhibits autophagy by attenuating the ROS production^22^. As Gln metabolism is essential for maintaining cellular redox homeostasis, in part via supporting the production of glutathione^23^, we probed whether enhanced Gln anaplerosis inhibits autophagy by regulating ROS. However, we found that DMKG treatment did not significantly affect cellular ROS production in 8988T cells (Fig. 4a). Moreover, even the presence of exogenous hydrogen peroxide (H_2_O_2_), addition of DMKG attenuates autophagy levels (Fig. 4b), indicating that DMKG can regulate autophagy expression in a ROS-independent manner.

Mammalian TORC1 (mTORC1) has a pivotal role in regulating autophagy^24^ and accruing evidence suggest that Gln metabolism pathways are important regulator of mTORC1 signaling^20^,^25^. Because upregulation of the intracellular level of αKG by glutaminolysis stimulates mTORC1 activation^20^, we next considered the possibility that Gln anaplerosis reduces autophagy through mTORC1 activation in PDAC cells. To test this idea we first examined the effect of DMKG on mTORC1 activity. Consistent with previous work, we observed that DMKG treatment markedly increased mTORC1 signaling, as evidenced by enhanced phosphorylation of S6K (Fig. 4c). Under these conditions, we did not detect comparable changes in endogenous levels of total mTOR (Supplementary Fig. 4).

Next, to probe further whether the inhibition of mTORC1 restored autophagy in DMKG treated 8988T cells, we cultured cells with mTORC1 inhibitors including LY294002 and rapamycin. The inhibition of mTORC1 pathway correlated with an increase in autophagy, restoring its expression to untreated levels (Fig. 4e). In addition, we observed similar results by measuring the aggregation of GFP-LC3 puncta (Fig. 4d). Taken together, these data clearly demonstrate that mitochondrial Gln anaplerosis inhibits autophagy by activating mTORC1 in PDAC cells.

## Discussion

In this study we demonstrate that Gln anaplerosis regulates PDAC growth and survival through autophagy. Previously, it has been shown that many cancer cells rely on an enhanced Gln anaplerosis to sustain their growth. Our study reveals that PDAC cells exhibit high vulnerability to mitochondrial Gln metabolism. Genetic and pharmacological increase of Gln anaplerosis attenuates PDAC growth and survival (Figs 1 and 2). We find that elevated mitochondrial carbon flow in PDAC cells leads to a decrease of cellular autophagy (Fig. 3) which is essential for their growth and survival. This idea is further validated by the finding that upregulation of Gln anaplerosis inhibits autophagy via regulation of mTORC1 signaling in PDAC (Fig. 4).

Recent studies have shed light on the mechanism through which Gln metabolism regulates cellular autophagy. Enhanced glutaminolysis and αKG production induce mTORC1 activation by stimulating GTP loading of RagB and lysosomal translocation, which inhibits autophagy^20^. Additionally, it was also shown that knockdown of GDH activity stimulates autophagy by inhibiting mTORC1 activation^22^. Consistent with these results, we found that enhanced Gln anaplerosis by DMKG treatment or GDH overexpression limits basal autophagy formation in PDAC cells through mTORC1 activation. ROS-mediated regulation of autophagy has also been suggested. Inhibition of autophagy resulted in an increase of cellular ROS levels and, conversely, antioxidant treatment decreased autophagy in PDAC^15^. Thus, it was proposed that the elevated basal autophagy is required for PDAC growth by preventing the accumulation of ROS^15^. Although DMKG treatment slightly decreased cellular ROS production, we did not find a significant role of ROS on autophagy regulation by Gln anaplerosis under the conditions used in our analysis (Fig. 4).

Interestingly, one recent paper showed that cytosolic acetyl-coenzyme A (AcCoA) functions as a key regulator of cellular autophagy^26^. An elevation of intracellular AcCoA increases the activity of the acetyltransferase EP300, which might inhibits autophagy in an mTORC1 dependent manner^26^. As Gln-derived αKG contributes to the production of citrate by undergoing reductive carboxylation via isocitrate dehydrogenase^26^,^27^, mitochondrial citrate derived from this pathway can be used for the synthesis of cytosolic AcCoA. Thus, it is possible that enhanced Gln anaplerosis inhibits autophagy in PDAC via the regulation of cytosolic AcCoA levels. However, because αKG can directly stimulate lysosomal translocation and activation of mTORC1^20^, the involvement of AcCoA in PDAC growth requires further investigation.

In comparison with other cancer cells, PDAC cells exhibit several distinct properties. They show an elevated autophagy under basal conditions and have a dependence on a non-canonical Gln pathway^11^,^15^. As autophagy is required for tumorigenic growth of PDAC^15^ and an increased Gln metabolism leads to the repression of autophagy through hyper-activation of mTORC1 signaling^20^, PDAC might need to limit carbon flux into TCA cycle by exporting Gln-derived aspartate into the cytoplasm via transcriptional regulation of key metabolic enzymes. Thus, this may explain why PDAC cells are markedly sensitive to enhanced Gln anaplerosis such as DMKG treatment or GDH overexpression.

PDAC exhibits pronounced resistance to various drugs^4^,^5^. Because the upregulation of autophagy supports chemotherapeutic resistance of cancer cells^28^, it was suggested that the elevated autophagy may contribute to the therapeutic resistance of PDAC. Our study demonstrates that increased Gln anaplerosis markedly represses autophagy formation, which inhibits tumorigenic growth of PDAC. Therefore, we speculate that these findings may have important implications for developing therapeutic approaches for PDAC. Given that clinical-grade Gln anaplerosis regulators are being developed^29^, the regulation of this pathway can potentially synergize with standard PDAC therapies, such as chemotherapy and radiation.

## Methods

The methods were carried out in accordance with the approved guidelines.

### Cell Culture

8988T and other human PDAC cell lines (kindly provided by Dr. Alec C. Kimmelman, Dana-Farber Cancer Institute (DFCI), Boston, MA, USA) were cultured in Dulbecco’s modified Eagle’s medium (Invitrogen, Grand Island, NY, USA) supplemented with 10% fetal bovine serum (HyClone, Logan, UT, USA) and penicillin/streptomycin (Invitrogen). The human GDH was cloned into retroviral pBabe vector and used to generate stable cell lines.

### Western Blotting

Cells were lysed with lysis buffer (150 mM NaCl, 50 mM Tris–HCl, pH 7.5 and 0.5% NP-40) supplemented with protease inhibitor cocktail (Roche, South San Francisco, CA, USA) and phosphatase inhibitor (Sigma, St Louis, MO, USA). Cell lysates were separated by sodium dodecyl sulfate-polyacrylamide gel electrophoresis and immunoblotting. The following antibodies were used: LC3 (Cell signaling, Danvers, MA, USA), S6K1-P (Cell signaling), S6K (Cell signaling) and β-actin (Sigma).

### ROS determination

The levels of cellular and mitochondrial superoxide were assessed by using DCFDA (Invitrogen) according to the manufacturer’s instructions. Briefly, cells were washed with phosphate-buffered saline and labeled at 37 °C for 20 min in Hank’s balanced salt solution (Gibco, Big Cabin, OK, USA) containing 10 μM DCFDA. Cells were trypsinized and resuspended in Hank’s balanced salt solution. Fluorescence was measured by flow cytometry using a FACSCalibur (BD Biosciences, San Jose, CA, USA).

### Flow Cytometric Measurement

Cells at less than 80% confluence were treated with DNA damage agents. After treatment, cells were harvested by trypsinization, pelleted by centrifugation, and resuspended in PBS containing 3% fetal bovine serum. The measurement of cell death was performed by flow cytometry using propidiumiodide (PI) staining, as previously described^18^. The apoptotic cell death was measured with AnnexinV-FITC (BD Pharmingen) according to the manufacturer’s instructions.

### Cell viability assay

PDAC cell lines were plated into 96-well plates at 1000 cells per well in 100 μl of growth media. The following day, growth media was replaced with that containing DMKG. Parallel plates were analyzed at 3 days by Cell Titer Glo analysis (Promega, Fitchburg, WI, USA), per the manufacturer’s instruction.

### Electron microscopy

LC3-GFP Transfected 8988T cells were grown on coverslips in a 6-well plate with DMSO, DMKG, DMKG/rapamycin or DMKG/LY294002. The cells were fixed in 4% paraformaldehyde for 20 min at room temperature and were washed in PBS twice. After washing, slides were mounted with Vectashield mounting medium with DAPI (Vector Laboratories, Burlingame, CA, USA). The fluorescence signal was detected using confocal microscopy or Personal DeltaVision.

### Clonogenic assay

Cells were plated in 6-well plates at 200 cells per well in 2 ml of growth medium. After 7–10 days, cells were fixed in 80% methanol and stained with 0.2% crystal violet and colonies were counted. The surviving fraction was calculated using the plating efficiency. Media was not changed throughout the course of experiment.

### Soft agar assay

Anchorage-independent growth was assessed by plating cells in a two-layer agar system (Millipore, Billerica, MA, USA), in which the final concentration of the bottom agarose layer was 0.8% and 0.4% for the top agarose layer that contained the cells.

### Statistical Analysis

Unpaired two-tailed Student’s *t* test was performed unless otherwise noted. All experiments were performed at least two or three times.

## Additional Information

**How to cite this article**: Jeong, S. M. *et al*. Enhanced mitochondrial glutamine anaplerosis suppresses pancreatic cancer growth through autophagy inhibition. *Sci. Rep.* **6**, 30767; doi: 10.1038/srep30767 (2016).

## Supplementary Material

Supplementary Information
