# Supplementary material for: Enhanced mitochondrial glutamine anaplerosis suppresses pancreatic cancer growth through autophagy inhibition
Source: Sci Rep. 2016 Aug 1;6:30767. doi: 10.1038/srep30767 (PMC4967856; doi:10.1038/srep30767)
Supplement: Supplementary Information [file srep30767-s1.pdf]

Supplementary Information

Enhanced mitochondrial glutamine anaplerosis suppresses pancreatic cancer growth through autophagy inhibition

Seung Min Jeong, Sunsook Hwang, Kyungsoo Park, Seungyeon Yang and Rho Hyun Seong

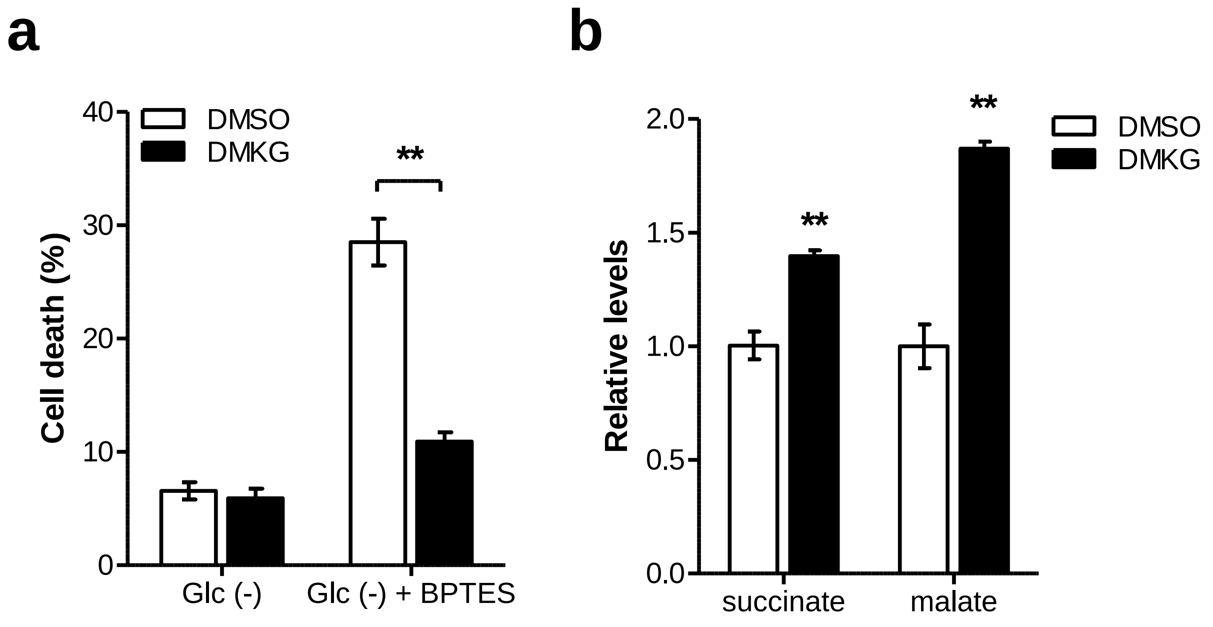

Supplementary Figure S1. DMKG treatment enhances mitochondrial Gln anaplerosis. (a) Cell viability of DMSO or DMKG (5 mM) treated 8988T cells deprived of glucose and supplemented with or without BPTES (5 μM). Cell viability was measured via PI exclusion assay. (b) Relative levels of succinate and malate in 8988T cells treated with DMKG (5 mM) when compared with DMSO treated control cells. Metabolites were extracted in ice-cold methanol (80 %), and endogenous metabolites were obtained using High-performance liquid chromatography (HPLC) with ultraviolet and refractive index detectors. Metabolites levels were normalized to protein content. All error bars ±SEM. $^{**}p < 0.01$.

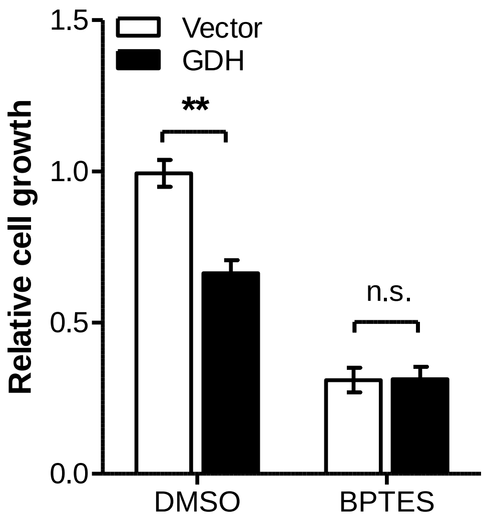

Supplementary Figure S2. Mitochondrial Gln anaplerosis regulates PDAC growth. Relative proliferation of control (Vector) and GDH-overexpressed (GDH) 8988T cells treated with or without BPTES (5 μM). All error bars ±SEM. n.s., not significant. **$p < 0.01$.

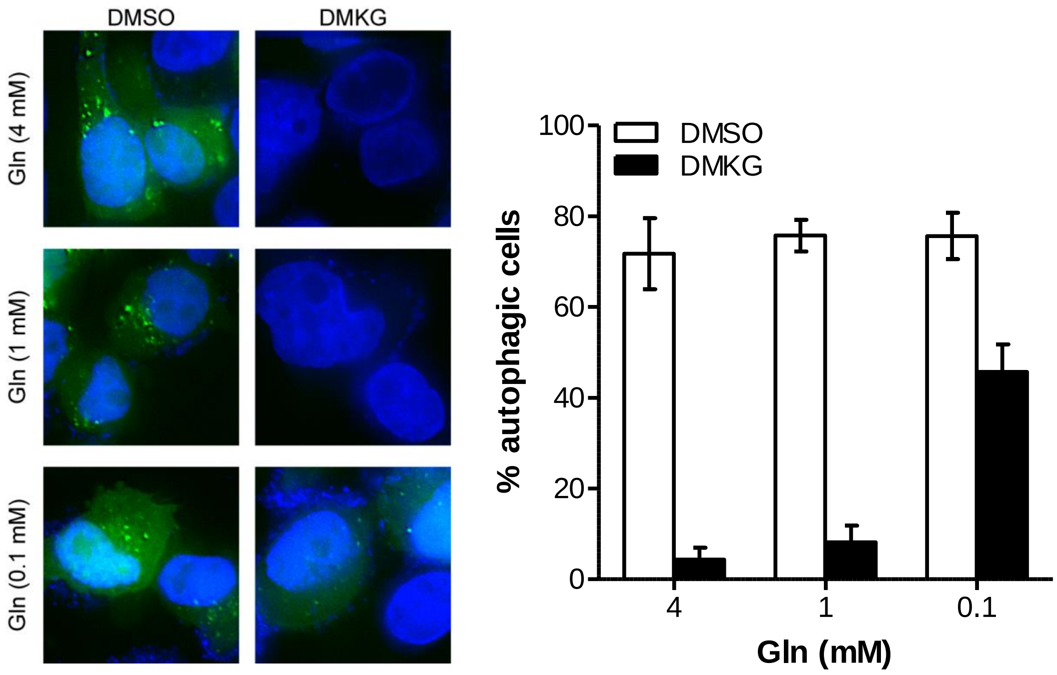

Supplementary Figure S3. Aggregation of GFP-LC3 in 8988T cells treated with DMKG (5 mM) under indicated Gln concentrations. Representative images of GFP-LC3 in 8988T cells cultured in complete (4 mM) or low glutamine (1 or 0.1 mM) medium treated with or without DMKG (left). The percentage of autophagic cells (defined as the presence of more than five autophagy foci) was quantified (right).

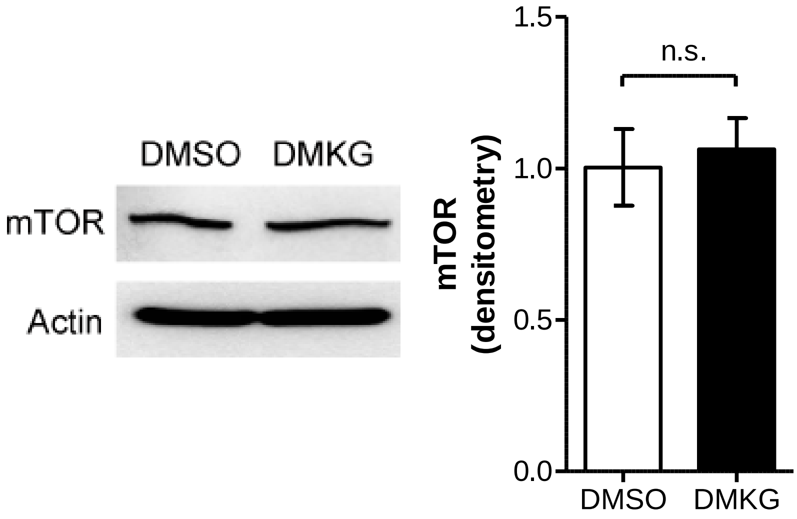

Supplementary Figure S4. Immunoblot analysis of total mTOR protein levels in whole-cell lysates from 8988T cells treated with or without DMKG (5 mM). β-actin serves as a loading control. n.s., not significant.
